# *Helicobacter pylori*-induced STAT3 activation and signalling network in gastric cancer

**DOI:** 10.18632/oncoscience.62

**Published:** 2014-07-03

**Authors:** Junhong Zhao, Yujuan Dong, Wei Kang, Minnie Y. Go, Joanna HM. Tong, Enders KW. Ng, Philip WY. Chiu, Alfred SL. Cheng, Ka Fai To, Joseph JY. Sung, Jun Yu

**Affiliations:** ^1^ Institute of Digestive Disease and Department of Medicine and Therapeutics, State Key Laboratory of Digestive Disease, Li Ka Shing Institute of Health Sciences, The Chinese University of Hong Kong, Hong Kong, China; ^2^ Department of Anatomical and Cellular Pathology, The Chinese University of Hong Kong, Hong Kong, China; ^3^ Department of Surgery, The Chinese University of Hong Kong, Hong Kong, China

**Keywords:** *Helicobacter pylori*, STAT3, gastric carcinogenesis, molecular regulator

## Abstract

**Background:**

*Helicobacter pylori (H. pylori)* is the most important gastric carcinogen. However, the mechanisms of *H. pylori* induced gastric carcinogenesis through STAT3 activation are largely unknown. We evaluated the effects of *H. pylori* infection on STAT3 activation and dissected the signalling network of STAT3 in *H. pylori-* infected gastric carcinogenesis.

**Methods:**

The expression of phospho-STAT3 (pSTAT3) was evaluated by immunohistochemistry and western blot. Gene expression array and chromatin immunoprecipitation were used to dissect the STAT3 signalling network on *H. pylori* co-cultured AGS.

**Results:**

pSTAT3 was significantly higher in *H. pylori* -positive gastritis than in *H. pylori* -negative gastritis ( *P* = 0.003). In addition, 98% of *H. pylori* positive intestinal metaplasia specimens showed STAT3 activation, whereas pSTAT3 was significantly decreased in all 43 specimens one year after *H. pylori* eradication ( *P* < 0.001). Moreover, pSTAT3 was only detected in the *H. pylori* -infected gastric tissues of mice but not in control mice. We further identified 6 candidates ( *BRUNOL4*, *FGFR1*, *SHOX2*, *JAK3*, *MAPK8*, and *PDPN* ) were directly up-regulated by *H. pylori* induced STAT3 activation.

**Conclusion:**

*H. pylori* infection triggers the activation of STAT3 and de-regulates multitude of tumorigenic genes which may contribute to the initiation and progression of gastric cancer.

## INTRODUCTION

Infection with the gram-negative bacterium *Helicobacter pylori* (*H. pylori*) is a major risk factor for gastric carcinoma. The cytotoxin-associated antigen A (CagA) gene codes for one of the *H. pylori* virulence proteins. Epidemiological studies reveal that CagA positive *H. pylori* strains are most closely related with an increased risk of gastric carcinogenesis in comparison with *H. pylori* CagA negative strains [1, 2]. The CagA+ *H. pylori* strains infection could increase signal transducer and activator of transcription 3 (STAT3) and mitogen-activated protein kinase (ERK1/2) activation in *H. pylori*-dependent gastritis [3]. It has been also showed that activation of STAT3 pathway is associated with progression to gastric cancer [4]. These findings suggest that *H. pylori* infection interfere with STAT3 proteins activation in gastric epithelial cells, which may favour their long term colonization in the host stomach.

STAT3, a cytoplasmic signalling protein and nuclear transcription factor, is frequently found to be over-activated in a variety of human malignancies. Persistent STAT3 signalling could promote the growth and survival of tumor cells, induce tumor angiogenesis and suppress the anti-tumor immune responses [5, 6]. Targeted deletion of STAT3 was shown to prevent epithelial cancer [7] and may be a novel approach in cancer therapy [8]. Constitutive activation of STAT3 has also been demonstrated in human gastric cancer cell lines and in primary human gastric cancers [3, 9]. STAT3 expression strongly correlated with VEGF expression and microvessel density in human gastric cancer [10]. Moreover, transfection of dominant negative STAT3 or inhibition of STAT3 by AG490 in human gastric cancer cell lines resulted in reduced cell growth [9]. These data suggest an important role of STAT3 in the pathogenesis of human gastric cancer. However, the mechanisms of *H. pylori induced* STAT3 signalling network in gastric carcinogenesis are still largely unknown. In this study, we aim to evaluate the effect of *H. pylori* infection on STAT3 activation and to dissect the signalling network of STAT3 in the *H. pylori-*infected gastric carcinogenesis.

## RESULTS

### STAT3 was activated in H. pylori-infected AGS cells

**Figure 1:**
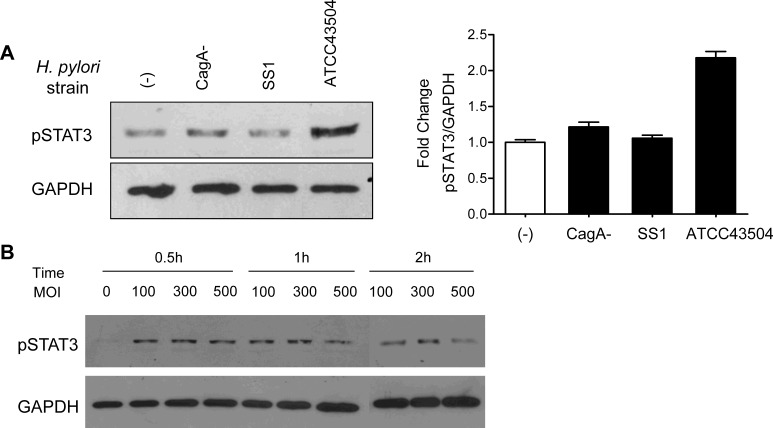
STAT3 was activated in H. pylori ATCC43504 infected AGS cells (A) AGS cells were treated with CagA+ *H. pylori* strain ATCC43504 and CagA-*H. pylori* strains respectively. Whole cell lysates were analysed by immunoblotting with anti-pSTAT3 (Tyr705) antibody. The pSTAT3 protein band intensities were quantified and normalized to GAPDH intensities (right panel). (B) The expression of pSTAT3 was induced in AGS after treatment with ATCC43504 at various MOI and co-culture time.

To elucidate whether infection of virulence *H. pylori* trigers the STAT3 activation in GC cells *in vitro*, we co-cultured the GC cell lines AGS with CagA+ *H. pylori* strain and CagA-*H. pylori* strains respectively. Remarkably, treatment of AGS cells with CagA+ ATCC43504, but not the isogenic CagA mutant strains, resulted in a pronounced increasing in phosphorylation of STAT3 (pSTAT3) at Tyr705 (Figure 1A). Therefore, AGS and ATCC435040 were used for further experiments.

We next performed a time/*H. pylori* MOI course study to monitor the pSTAT3 level at different time points after *H. pylori* ATCC43504 infection. We observed that *H. pylori* increased the pSTAT3 by nearly 2-fold at 0.5 hr in AGS cells; the pSTAT3 level was regulated in a time-dependent manner (Figure 1B). However, we did not observe a clear difference on the pSTAT3 level in response to different *H. pylori* MOIs (Figure 1B). Taken together, we established *H. pylor*i strain ATCC43504 and AGS co-culture model. Our findings demonstrated that pSTAT3 was induced by CagA positive *H. pylori* in AGS.

### pSTAT3 was increased in H. pylori-associated gastric tissues

**Figure 2:**
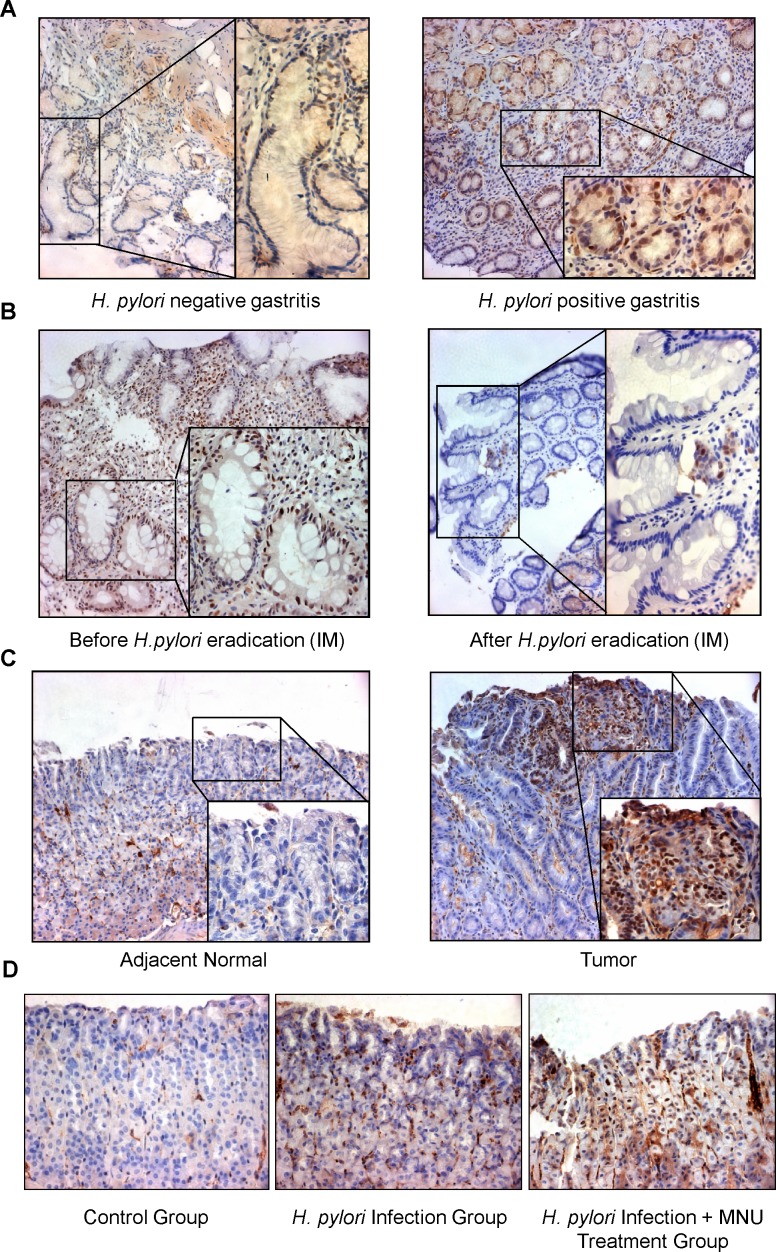
pSTAT3 was increased in H. pylori-associated clinical gastric tissues (A) Representative images of immunohistochemical staining of pSTAT3 in *H. pylori* negative gastritis*,* and in *H. pylori* positive cases. (B) Representative images of immunohistochemical staining of pSTAT3 in *H. pylori* infected intestinal metaplasia (IM) before and one year after eradication. (C) Representative images of immunohistochemical staining of pSTAT3 in gastric cancer tumor tissues and adjacent normal tissues. Original magnification, ×200 (low power); ×400 (high power). (D) pSTAT3 was increased in *H. pylori* SS1 infection mouse model. Representative images of immunohistochemical staining of pSTAT3 in *H. pylori* SS1 infection mouse model with or without MNU treatment. Original magnification, ×200.

To investigate the effect of *H. pylori* on STAT3 activation in human gastric tissues, we examined the changes in pSTAT3 protein level on clinical specimen using immunohistochemistry (Figure 2A). The pSTAT3 staining analysis was summarized in Table 1. We found that the expression of the active form of STAT3 was significantly higher in *H. pylori*-positive gastritis (73.5%, 61/83) than in *H. pylori*-negative gastritis (50%, 35/70) (*P* = 0.003). To further confirm the connection between *H. pylori* infection and STAT3 activation, we measured the pSTAT3 level on 44 paired gastric intestinal metaplasia biopsies taken from *H. pylori* infected individuals before and one year after *H. pylori* eradication. We found that 98% (43/44) of *H. pylori* positive intestinal metaplasia specimens showed positive staining for pSTAT3 before eradication, whereas therapy-based eradication of *H. pylori* significantly decreased pSTAT3 expression in all the 43 *H. pylori*-positive intestinal metaplasia patients (Table 2, Figure 2B, P < 0.001). These data provided direct evidence that *H. pylori* contributed to the activation of STAT3 in gastritis and intestinal metaplasia. In addition, pSTAT3 expression was detected in 27 human *H. pylori* positive gastric cancers using immunohistochemistry. pSTAT3 displayed stronger nuclear staining in the gastric cancer specimens compared with the adjacent normal tissues (Figure 2C).

**Table 1:** Expression of pSTAT3 in H. pylori negative and H. pylori positive gastritis

	No.	pSTAT3		χ^2^	*P* Value
		Negative	Positive		
*H. pylori* negative gastritis	70	35 (50%)	35 (50%)		
*H. pylori* positive gastritis	83	22 (26.5%)	61 (73.5%)	8.97	0.003

**Table 2:** pSTAT3 immunohistochemistry in 44 cases with paired intestinal metaplasia gastritis before and after H. pylori eradication

	No.	pSTAT3		χ^2^	*P* Value
		Negative	Positive		
Before	44	1 (2.3%)	43 (97.7%)	84.09	<0.001
After	44	44 (100%)	0 (0%)		

### The expression of pSTAT3 was increased in H. pylori infection mouse model

The association between *H. pylori* infection and pSTAT3 expression was further evaluated in mouse model with or without *H. pylori* Sydney Strain 1 (SS1) infection. We found that pSTAT3 was only detected in the *H. pylori*-infected gastric tissues of mice (2/3, 66.7%), but not in those of the control mice (Table 3), providing further evidence that *H. pylori* infection stimulated the activation of STAT3 in the stomach (Figure 2D). Moreover, treatment of *H. pylori* infected mice with N-methyl-N-nitrosourea (MNU), an alkylating agent that induces gastric carcinogenesis in mouse, resulted a pronounced STAT3 activation in all the mice tested (Table 3, 100%, *P* = 0.005). Thus, this *in vivo* study recapitulated our cellular and clinical findings, and demonstrated directly that *H. pylori* induced STAT3 activation which contributing to the gastric carcinogenesis.

**Table 3:** Expression of pSTAT3 in H. pylori associated mouse model

	No.	pSTAT3		χ^2^	*P* Value
		Negative	Positive		
Control group	4	4 (100%)	0 (0%)		
*H. pylori* infection group	3	1 (33.3%)	2 (66.7%)	3.733	0.053
*H. pylori* infection + MNU treatment group	4	0 (0%)	4 (100%)	8.0	0.005

### STAT3 promoted GC cell growth

**Figure 3:**
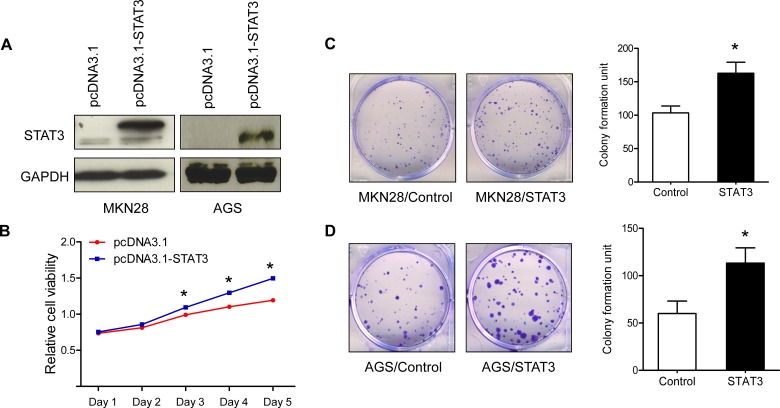
STAT3 processes oncogenic function in gastric cancer cells (A) Ectopic expression STAT3 in AGS and MKN28. (B) Ectopic expression STAT3 promoted MKN28 cell growth. (C) Colony formation assay of STAT3-expressing or control MKN28 cells. (D) Colony formation assay of STAT3-expressing or control AGS cells Representative images of colonies formed are shown. * P < 0.05.

The activation of STAT3 after *H. pylori* infection led us to speculate that enhanced STAT3 activation contributed to the proliferative phenotype of the GC cells. Thus, we transfected pCDNA3.1-STAT3 into GC cell line MKN28 and AGS (Figure 3A) and cell variability was measured by MTT assay and colony formation assay. As expected, MKN28 cells with up-regulated STAT3 grew significantly faster than did control cells (Figure 3B). In addition, overexpression of STAT3 in AGS and MKN28 significantly promoted the colony growth (Figure 3C and 3D).

### Identification of STAT3 associated transcriptional target genes and pathways in H. pylori-infected AGS

**Figure 4:**
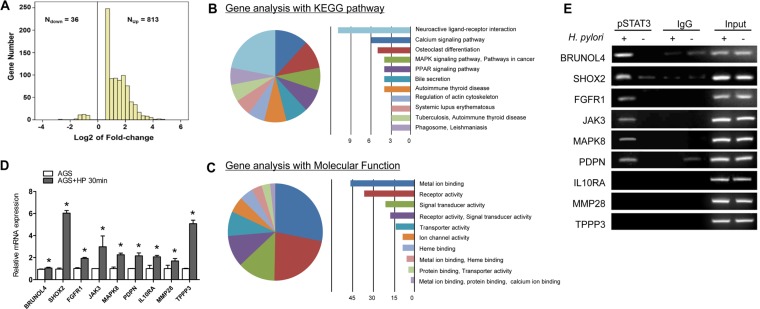
Identification of key tumorigenic-regulators directly controlled by STAT3 following H. pylori infection (A) 849 genes were differently expressed in ATCC43504-infected AGS relative to control group. (B) Top 11 dysregulated pathways using the KEGG database. (C) Gene ontology of the cDNA microarray. (D) Quantitative RT-PCR validation of the 9 potential targets in AGS cells treated with ATCC43504 for 30 min relative to the untreated cells. GAPDH was used as an internal control. (E) Confirmation of the potential pSTAT3 target genes by ChIP-PCR using anti-pSTAT3 (Tyr705) antibody or irrelevant antibody against IgG (negative control) on ATCC43504 infected and non-infected AGS cells. Input represents the genomic DNA. *P < 0.05.

To assess the *STAT3 associated* transcriptional alterations induced by *H. pylori* infection, we performed genome expression microarrays on the ATCC43504-infected and non-infected AGS. AGS cells treated with either ATCC43504 at 100 MOI or PBS for 0.5 hr were subjected to microarrays. Analysis of the microarray data for the two groups revealed a total of 849 genes with expression changes, among which 813 genes showed increased expressions in *H. pylori*-infected AGS with STAT3 activation and 36 genes showed decreased expressions (Figure 4A). These candidates with fold change ≥ 2 (53 candidates up-regulated in *H. pylori*-infected AGS) were subjected to gene ontology and pathway analysis. KEGG pathway analysis showed that the dysregulated genes were enriched in 11 different cancer pathways including ligand-receptor interaction, calcium signalling pathway, cell differentiation and MAPK signalling pathway (Figure 4B). Gene ontology analysis revealed significant up-regulation of genes with prominent roles in multiple cellular processes including ion binding, signal transduction and transporter activity (Figure 4C). Nine potential targets up-regulated in the expression microarray (*BRUNOL4*, *FGFR1*, *SHOX2*, *JAK3*, *MAPK8*, *PDPN*, *IL10RA*, *MMP28*, and *TPPP8*) were selected for further real-time PCR validation. We found that these candidates were significantly increased at transcriptional level upon infection of *H. pylori* in AGS (Figure 4D).

### Identification of direct STAT3 transcriptional target genes in H. pylori-infected AGS cells

To identify key tumorigenic-regulators directly controlled by STAT3 binding after *H. pylori* infection, we next performed ChIP on ATCC43504 infected AGS cells. After cross-linked with formaldehyde, DNA was sonicated into small fragments and immunoprecipitated by anti-pSTAT3 (Tyr705) polyclonal antibody. Conventional real-time PCR and ChIP-PCR analysis validated that 6 out of the 9 potential pSTAT3 (Tyr705) targets (*BRUNOL4*, *FGFR1*, *SHOX2*, *JAK3*, *MAPK8* and *PDPN*) showed strong enrichment for pSTAT3 antibody but not the IgG control (Figure 4E). Collectively, our data suggested that the 6 candidates could be the potential tumorigenic genes that directly regulated by activated STAT3 in response to *H. pylori* infection.

## DISCUSSION

In our study, we established a gastirc cancer cell line-*H. pylori* co-culture model and demonstrated that *H. pylori* pronouncedly induced STAT3 activation in GC cell line AGS after co-culture *in vitro*. By using a larger clinical sample cohort, we observed a correlation between STAT3 activation and *H. pylori* infection. We found an increased pSTAT3 level in the *H pylori* positive gastritis patients (Table 1). As shown in Table 2, we also found that STAT3 activation was significant reduced in the intestinal metaplasia one year after *H. pylori* eradication as compared with their initial *H. pylori* positive intestinal metaplasia specimen from 44 patients (*P* < 0.001). These data suggests that *H. pylori* infection and its related STAT3 activation play the pivotal role in the process of gastric inflammation to intestinal metaplasia, an important part of a progressive process of gastric cancer. Moreover, we found that pSTAT3 displayed strongly nuclear staining in the *H. pylori* positive gastric cancer specimen, whereas the pSTAT3 staining was relatively weak in the adjacent normal stomach. Thus, based on these clinical specimens, we found that pSTAT3 was significantly associated with *H. pylori*-positive gastritis, *H. pylori*-positive intestinal metaplasia and gastric cancer. In mice, we observed that expression of pSTAT3 was increased in response to *H. pylori* infection, and up-regulated remarkably in a MNU induced gastric cancer. Collectively, our findings suggested that STAT3 activation in response to *H. pylori* infetion contributed to the initiation of gastric carcinogenesis.

We also demonstrated the enhanced STAT3 expression contributed to the proliferative phenotype of the AGS and MKN28 cell lines. Upon activation, pSTAT3 translocates to cell nucleus and mediates the expression downstream effector genes, leading to cell survival, proliferation, angiogenesis, invasion and migration [11]. Using gene expression microarray, we identified that totally 849 genes were dysregulated in AGS after *H. pylori* infection. The dysregulated genes were enriched in 11 different cancer pathways including ligand-receptor interaction, calcium signalling pathway, cell differentiation and MAPK signalling pathway (Figure 4B) with an important implication for the *H. pylori*-induced gastric tumorigenesis (Figure 4C). Elucidating the direct downstream genes of STAT3 in response to *H. pylori* infection is important for unrevealing critical molecular events of gastric carcinogenesis. Using gene expression microarray coupled with ChIP-PCR assays, we identified 6 candidate genes (*BRUNOL4*, *FGFR1*, *SHOX2*, *JAK3*, *MAPK8*, and *PDPN*) that directly up-regulated by *H. pylori* induced STAT3 activation (Figure 4E). Among these candidates, *BRUNOL4* and *FGFR1* are the two novel oncogenic candidates that may play roles in gastric carcinogenesis. Bruno-like 4 splicing factor (BRUNOL4, also known as CELF4) belongs to the CELF/BRUNOL protein family which involved in post-transcriptional gene expression processes, including pre-mRNA alternative splicing and editing. [12]. Although the function of BRUNOL4 in gastric cancer development is still largely unknown, Rouzier reported that the expression of BRUNOL4 was associated with paclitaxel sensitivity in breast cancer [13]. FGFR1 is a member of the fibroblast growth factor receptor family, which interacts with its ligand fibroblast growth factors (FGF) to induce a cascade of downstream signalling that regulates cell proliferation, survival, migration and differentiation [14]. The potent oncogenic potential of the FGFR1 has been confirmed by transgenic mouse study where Fgfr1 express in the mammary epithelium, resulting in enhanced mammary tumorigenesis [15]. In human, gene amplification of FGFR1 in 22% of squamous cell lung cancer [16], and 10% of ER-positive breast cancer [17].

In conclusion, our results indicated that *H. pylori* infection triggers the activation of STAT3 to de-regulate a multitude of tumorigenic genes which may contribute to the initiation and transformation of gastric carcinogenesis through mediating the downstream effectors and signalling pathways of STAT3.

## METHODS

### Cell culture

The gastric cancer cell lines AGS and MKN28 were purchased from the American Type Culture Collection (ATCC, Manassas, VA) and maintained in RPMI 1640 growth medium, supplied with 10% fetal bovine serum, 50 IU/mL penicillin, 50 ug/mL streptomycin (All Invitrogen, Carlsbad, CA). Cells were kept at 37°C in a humidified incubator with 5% CO_2_.

### *H. pylori* culture and in vitro infection model

*H. pylori* strains (SS1, CagA-, ATCC43504 CagA+) were inoculated on Brucella agar plates containing 5% sheep blood and incubated at 37 °C for 3-4 days under microaerophilic conditions in a humidified CO2 incubator (Thermo Fisher Scientific, Wilmington, DE). The plate culture was then suspended and *H. pylori* were incubated with gastric cancer cells for the indicated incubation period (0.5, 1, and 2 hr). The cells were then washed three times with PBS and harvested.

### Patients and gastric biopsies

Gastric biopsies were collected from primary *H. pylori* associated chronic gastritis (83 cases) and gastric cancer (27 cases) during endoscopy according to a standard protocol, prior to any therapeutic intervention. We also rescued 44 paired gastric biopsies taken from *H. pylori* infected individuals before and one year after eradication. Moreover, gastric biopsies were also collected from *H. pylori* negative gastritis (70 cases) as controls. Patients were excluded if they had other cancers or inflammation diseases. All patients and controls gave informed consent, and the study protocol was approved by the Clinical Research Ethics Committee of the Chinese University of Hong Kong.

### *H. pylori* infection in mouse model

A total of 11 male C57BL/6 mice (6-8-week old) were obtained from the Animal Service Centre at the Chinese University of Hong Kong. All of the mice were given a standard pellet chow diet and autoclaved distilled water *ad libitum* and were maintained in specific pathogen-free conditions. MNU (240 p.p.m. Sigma-Aldrich, St Louis, MO) was prepared freshly twice a week by dissolving in distilled water. The solutions were administered *ad libitum* for a total 5 cycles of 1-week regimen followed by 1-week pause, as drinking water in light-shielded bottles. The *H. pylori* SS1 were used to infect the mice. After 48 hr of fasting, 0.1 mL suspension of *H. pylori* containing 1 × 10^9^ colony-forming units (CFU) /mL were delivered to mice intragastrically, triple times every other day within a week. The mice were sacrificed 40 weeks after *H. pylori* infection. Animal studies were performed in accordance with guidelines approved by the Animal Experimentation Ethics Committee of the Chinese University of Hong Kong.

### Chromatin immunoprecipitation (ChIP)

ChIP was performed as described previously [18]. Briefly, 1 × 10^8^ AGS cells treated with ATCC43504 multiplicity of infection (MOI) of 100 in serum free RPMI1640 medium for 0.5 hr were crosslinked with 1% formaldehyde for 10 minutes at room temperature and quenched by glycine. After cell lysis, the chromatin was fragmented into 100 - 500 bp by Bioruptor Sonicator (Diagenode, Denville, NJ) and protein-DNA complexes were immunoprecipitated (IP) by 5 ug anti-pSTAT3 (Tyr 705) antibody (Cell Signaling Technology, Beverly, MA) or anti-IgG antibody (Sigma-Aldrich) Dynal magnetic bead (Invitrogen) mix on rotator at 4°C overnight. After washing and reversal of crosslinks, the IP and input DNA were purified and subjected to PCR.

### Gene expression microarray analysis

Gene expression profiles of AGS and *H. pylori* infected AGS cells were analyzed using Whole Human Genome Microarray Kit, 4x44K (Agilent Technologies, Signal intensities were analyzed using a GenePix 4000A scanner (Axon Instruments, Molecular Devices Crop., Palo Alto, CA). Array data were presented as log base 2-ratio of the Cy5/Cy3 signals.

### Conventional and quantitative ChIP-PCR assays

For target genes validation, PCR primers targeting a region within 150 bp of the putative binding site were designed to detect IP and input DNA. 2 uL IP and 2% input DNA were used as a template for conventional PCR assay. For quantitative ChIP-PCR, equal amounts of IP and diluted input DNA were used for Power SYBR Green– based detection (Applied Biosystems, Grand Island, NY).

### Western blot

Protein lysates from cell lines were prepared using protease inhibitor cocktail-containing (Roche, Basel, Switzerland) lysis buffer. Protein concentration was determined by the Bradford method (Bio-Rad Laboratories, Hercules, CA). Antibody-antigen complexes were detected using the ECL Plus Western Blotting Detection Reagents (GE Healthcare Piscataway, NJ). Primary antibodies used were rabbit anti-STAT3 (Cell Signaling Technology, Boston, MA), rabbit anti-pSTAT3- Try705 (Cell Signaling Technology), mouse anti-GAPDH (Santa Cruz Biotechnology, Santa Cruz, CA).

### Immuhistochemistry

Five um sections from formalin-fixed paraffin-embedded archive tissues were stained with polyclonal antibody against pSTAT3 (1:25). Antibody was incubated at room temperature for 2 hr, and chromogen development was performed using the universal HRP Multimer Ultraview Kit on Benchmark XL (Ventana Medical System, Oro Valley, AZ). The nuclear expression was assessed by a proportion score of the positive tumor cells and was categorized into negative (extensity < 10%), positive (extensity ≥ 10%). The scoring was independently assessed by 2 investigators.

### Colony formation assay and cell viability assay

GC cell lines were transfected with pcDNA3.0- STAT3 or pcDNA3.0 using lipofectamine 2000 (Invitrogen) and selected with G418 at 0.5 mg/ml (Calbiochem, Darmstadt, Germany) for 14 days. The resulting cells were then fixed with 70% ethanol and stained with 5% crystal violet, and individual colonies with more than 50 cells were counted. Cell viability was determined using Vybrant MTT Cell Proliferation Assay Kit (Invitrogen).

### Statistical Analysis

The results were expressed as mean ± standard deviation (SD). Mann-Whitney U test was performed to compare the variables of the two sample groups. The association between patient characteristics and pSTAT3 status was analyzed by the χ^2^ test. All analyses were performed using SAS for Windows software, version 9 (SAS Institute). All tests of statistical significance were two-sided. *P*-values less than 0.05 were considered statistically significant.
